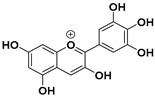# Current Perspectives of the Applications of Polyphenols and Flavonoids in Cancer Therapy

**DOI:** 10.3390/molecules25153342

**Published:** 2020-07-23

**Authors:** Xavier Montané, Oliwia Kowalczyk, Belen Reig-Vano, Anna Bajek, Krzysztof Roszkowski, Remigiusz Tomczyk, Wojciech Pawliszak, Marta Giamberini, Agnieszka Mocek-Płóciniak, Bartosz Tylkowski

**Affiliations:** 1 Department of Chemical Engineering, University Rovira i Virgili, Av. Països Catalans 26, Campus Sescelades, 43007 Tarragona, Spain; belen.reig@urv.cat (B.R.-V.); marta.giamberini@urv.cat (M.G.); 2 Research and Education Unit for Communication in Healthcare Department of Cardiac Surgery, Ludwik Rydygier Collegium Medicum in Bydgoszcz Nicolaus Copernicus University in Torun, M. Curie Sklodowskiej St. 9, 85-094 Bydgoszcz, Poland; oliwiakowalczyk111@gmail.com; 3 Kazimierz Wielki University, Jagiellonska St. 11, 95-067 Bydgoszcz, Poland; 4 Department of Tissue Engineering Chair of Urology, Ludwik Rydygier Collegium Medicum in Bydgoszcz Nicolaus Copernicus University in Torun, Karlowicza St. 24, 85-092 Bydgoszcz, Poland; a_bajek@wp.pl; 5 Department of Oncology, Nicolaus Copernicus University in Torun, Romanowskiej St. 2, 85-796 Bydgoszcz, Poland; roszkowskik@cm.umk.pl; 6 Department of Cardiac Surgery, Ludwik Rydygier Collegium Medicum in Bydgoszcz Nicolaus Copernicus University in Torun, M. Curie Sklodowskiej St. 9, 85-094 Bydgoszcz, Poland; r.tomczyk@wp.pl (R.T.); w.pawliszak@cm.umk.pl (W.P.); 7 Department of General and Environmental Microbiology, University of Life Sciences Poznan, ul. Szydłowska 50, 60-656 Poznań, Poland; agnieszka.mocek-plociniak@up.poznan.pl; 8 Eurecat, Centre Tecnològic de Catalunya. Chemical Technologies Unit, Marcel·lí Domingo s/n, 43007 Tarragona, Spain

**Keywords:** cancer, natural health products, phytochemicals, anticancer therapy, polyphenols, antioxidants, flavonoids, dietary supplements

## Abstract

The development of anticancer therapies that involve natural drugs has undergone exponential growth in recent years. Among the natural compounds that produce beneficial effects on human health, polyphenols have shown potential therapeutic applications in cancer due to their protective functions in plants, their use as food additives, and their excellent antioxidant properties. The possibility of combining conventional drugs—which are usually more aggressive than natural compounds—with polyphenols offers very valuable advantages such as the building of more efficient anticancer therapies with less side effects on human health. This review shows a wide range of trials in which polyphenolic compounds play a crucial role as anticancer medicines alone or in combination with other drugs at different stages of cancer: cancer initiation, promotion, and growth or progression. Moreover, the future directions in applications of various polyphenols in cancer therapy are emphasized.

## 1. Introduction

The appearance of the severe acute respiratory syndrome coronavirus 2 (SARS-CoV-2) in December last year and its very rapid spread around the world in early 2020, known to cause COVID-19 disease, has evidenced, among other things, the importance of investing in research to improve the people’s quality of life or eradicate diseases that still do not have an effective treatment.

One of the diseases for which possible therapies are still being studied is cancer. Cancer is known as a group of diseases that includes an unusual growth of malignant cells with the potential to invade or extend to other parts of the body [1,2]. The causes of cancer are strongly influenced by lifestyle and habits, which are fundamental when it comes to developing certain diseases such as obesity and heart disease, in addition to cancer. Some of these factors are smoking, obesity, processed meat consumption, radiation, family history, stress, environmental factors, etc. A scientific paper published in the journal “CA: A Cancer Journal for Clinicians” of the American Cancer Society estimated that during the year 2019, there were 1,762,450 new cancer cases diagnosed and 606,880 cancer deaths only in the United States [3], making cancer the second leading cause of death in the country. Moreover, the World Health Organization estimates that “deaths from cancer worldwide are projected to reach over 13 million in 2030” [4]. These two pieces of evidence demonstrate that cancer is one of the most severe health issues in the world.

Research has allowed a huge development in the prevention, detection, and treatment of cancer, leading to a decrease in mortality rates. Despite this, assigning the appropriate therapy for each type of cancer is still difficult today due to the late-stage diagnosis, inadequate strategies for addressing aggressive metastases, and a lack of clinical procedures for overcoming multidrug-resistant cancer [5].

The use of conventional medicines and procedures to treat tumors such as chemotherapy and radiation often results in the occurrence of deleterious side effects. Therefore, one of the current goals of cancer research is associated with the development of new therapies that are less harmful to the human body. On this path, natural compounds can be very useful [6,7].

Humans have always been able to extract infinite resources from nature. Furthermore, researchers try to mimic the observed natural models in order to develop useful tools and biomaterials [8].

Nowadays, a large number of natural resources have revealed high medicinal potential as exceptional candidates for the treatment of different types of diseases. Natural medicines can be obtained from different resources [9]:

- Vegetal
- Microbial
- Marine species.

Out of the resources mentioned above, natural compounds extracted from plants or phytochemicals have been used for centuries in traditional medicine. Phytochemicals are chemical compounds produced by plants that are typically involved in plant growth or in the process of protecting them from predators or pathogens [10,11]. Currently, the use of phytochemicals, especially polyphenols, as alternative anticancer drugs is a promising alternative, since they minimize or suppress the adverse effects of the usually more aggressive conventional therapies. Besides, our body develops resistance to certain conventional drugs involved in cancer therapy [12].

Therefore, this review presents a wide range of polyphenols that have been investigated in terms of application in different types of cancer therapies.

## 2. Research Methodology

A systematic search was performed to identify all relevant research papers published on the use of different polyphenols and their major subgroups as a potent anticancer treatment using the Web of Sciences database (1988–present). The search strategy was performed using several keywords to track down the relevant and more recent research articles, including ‘polyphenols in cancer’, ‘flavonoids in cancer’, ‘stilbenes in cancer’, ‘resveratrol in cancer’, ‘curcuminoids in cancer’, ‘curcumin in cancer’, ‘lignans in cancer’, ‘arctigenin in cancer’, ‘magnolol in cancer’, ‘honokiol in cancer’, ‘phenolic acids in cancer’, ‘*p*-coumaric acid in cancer’, ‘flavonoids in cancer’, ‘flavonols in cancer’, ‘kaempferol in cancer’, ‘quercetin in cancer’, ‘flavones in cancer’, ‘apigenin in cancer’, ‘luteolin in cancer’, ‘flavonones in cancer’, ‘naringenin in cancer’, ‘hesperetin in cancer’, ‘flavanols in cancer’, ‘epigallocatechin gallate in cancer’, ‘(−)-epicatechin in cancer’, ‘isoflavones in cancer’, ‘genistein in cancer’, ‘daidzein in cancer’, ‘chalcones in cancer’, ‘ellagic acid in cancer’, ‘anthocyanidins in cancer’, and ‘delphinidin in cancer’.

## 3. Polyphenols

Polyphenols are secondary metabolites produced by plants that are characterized by the presence of numerous phenolic rings [13]. The main sources of polyphenols are berries, grapes, olive oil, cocoa, nuts, peanuts, and other fruits and vegetables, which contain up to 200–300 mg of polyphenols per 100 g fresh weight. Moreover, products manufactured from these fruits such as tea, wine, or beer also contain polyphenols in significant amounts. The number and characteristics of the phenolic groups are responsible for the particular properties of each class of polyphenols (biological, chemical, and physical properties) [14].

In plants, polyphenols present different roles:

- Some of them are essential for plant physiological functions.
- Participate in defense processes against situations of stress and various stimuli (water, light, etc.).

**Figure 1:**
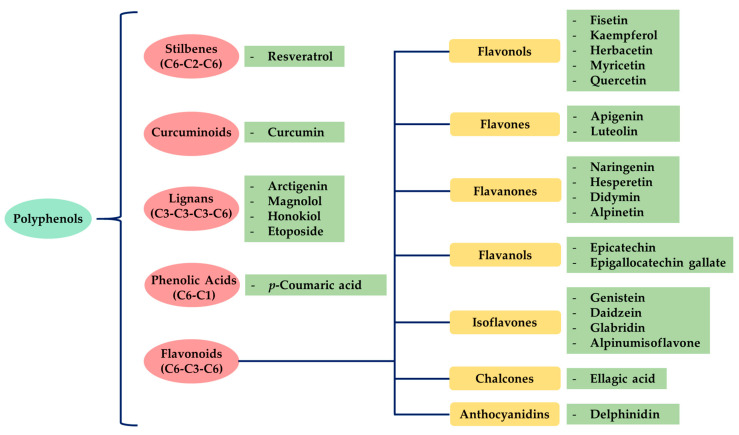
Classification of polyphenols and flavonoids. Examples of each subgroup with anticancer activity are mentioned.

There are several classes and subclasses of polyphenols, which have been established according to the number of phenolic rings and the structural elements of those rings. Figure 1 shows the classification of the main groups of polyphenols.

**Figure 2:**
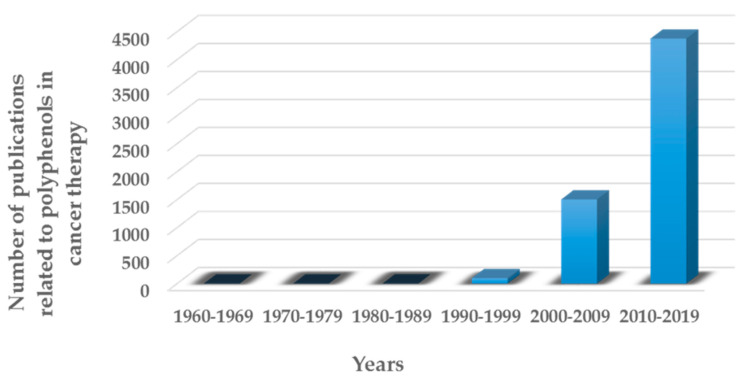
Number of peer-reviewed articles published in the last decades in the field of polyphenols in cancer therapy.

As observed in Figure 2, there has been an exponential increase of research and publications related to the possible use of polyphenolic compounds in cancer therapy [15]. The fact that polyphenols can be extracted using simple and green techniques—such as ultrasound-assisted extraction, and that after being sterilized, polyphenols preserve most of their properties intact—will contribute to the study of these compounds as potential anticancer drugs [16,17].

### 3.1. Stilbenes

Stilbenes or stilbenoids are hydroxylated derivatives of stilbene with a C6–C2–C6 chemical structure. These kinds of compounds are produced in various plants such as strawberries, grapes, peanuts, and cannabis [18]. Furthermore, various trees synthesize stilbenes as secondary products of heartwood that can act as antimicrobial and antioxidative substances. Stilbenes share most of their biosynthesis pathway with chalcones, which is a class of flavonoids.

The most representative compound of the stilbene family that has many health benefits is resveratrol [19].

#### Resveratrol

Resveratrol (3,5,4′-trihydroxy-trans-stilbene) is a natural polyphenol of the stilbene family. Resveratrol is produced by several plants (grapes, almonds, beans, blueberries, raspberries, mulberries, peanuts, etc.) in response to infections and injuries or as a defense against different kinds of pathogens attacks, such as fungi or bacteria [20]. Furthermore, red wine also contains significant amounts of resveratrol.

In 1997, *Jang* et al. were the first researchers that reported the inhibition of skin cancer development in mice by using resveratrol [19]. Since then, many investigations have suggested that resveratrol is able to prevent cancer or delay its onset [21].

In point of fact, studies demonstrated that resveratrol has in vitro effects against a large range of human tumors: breast, skin, ovary, stomach, prostate, colon, liver, pancreas, cervix, thyroid carcinoma cells, lymphoid, and myeloid cancer cells [22].

It has been proven that resveratrol shows beneficial effects at different stages of cancer (initiation, promotion, and progression of cancer). For example, resveratrol protects DNA from reactive oxygen species (ROS) and traps hydroxyls, superoxides, and free radicals produced in cells—events that are usually related to the initiation of tumors [23].

In another study, *Yin* et al. demonstrated that the application of resveratrol inhibits the promotion and progression of A549 lung cancer cells in nude mice. However, the authors mentioned that further studies should be performed in order to evaluate other parameters, such as the applied dose of resveratrol [24].

Besides, clinical trials on humans have been performed with the use of resveratrol, obtaining satisfactory results [25,26,27].

### 3.2. Curcuminoids

Curcuminoids are natural polyphenols that contain two phenol units joined through a linear diarylheptanoid. The presence of curcuminoids gives a yellow color to plants that contain these kinds of natural structures.

The phenolic rings of curcuminoids are chemically modified with other chemical groups with the aim of overcoming some drawbacks of natural curcuminoids in clinical applications such as their poor solubility, low absorption, and bioavailability [28]. Among the curcuminoids, curcumin is one of the most known and studied structures with a high potential as medicine to treat different cancers, apart from also being useful in treating other types of diseases. Nonetheless, the poor solubility of curcumin in water of acidic and physiological pH requires the use of diverse alternatives to avoid losing the effectiveness of curcumin as a medicine, such as the synthesis of other curcumin derivatives or the combination of curcuminoids with surfactants or co-surfactants.

#### Curcumin

Curcumin is a natural compound and the principal curcuminoid of turmeric plants, which is responsible for turmeric’s yellow color [29].

In addition to its applications in medicine, the use of curcumin has reached other fields. In the food industry, it has been used as a dietary supplement (it is sold as herbal supplement) or a food additive. Additionally, it is used in cosmetics and other products.

Curcumin is commonly used in cancer therapies of different types of cancer: lung, cervix, prostate, breast, bone, and liver [30]. Nevertheless, the administration of free curcumin presents some drawbacks: poor solubility in water, instability in aqueous conditions, low bioavailability, and poor cellular uptake. To overcome these problems, two different solutions were attempted:

- The synthesis of curcumin derivatives [31], and
- The encapsulation of curcumin in different nanostructures ranging from liposomes to natural biopolymeric nanoparticles [32,33].

One of the curcumin derivatives used in breast and renal cancer therapies is dimethoxy curcumin. Chen et al. recently proved that this curcumin derivative can be effective in the therapy of colon cancer cells due to causing the reduction of survivin expression and the enhancement of E-cadherin, a cell adhesion molecule, whose loss contributes to the formation of epithelial types of cancers such as carcinomas [34].

Recently, various research groups have reported that the combination of both curcumin and resveratrol can reduce the incidence of lung and prostate cancer [35,36].

### 3.3. Lignans

Lignans are diphenolic compounds found in a wide variety of plants including broccoli, beans, soybeans, rye, sesame seeds, pumpkin seeds, flax seeds, and some berries in very small amounts (μg of lignans per 1 g of dry product) [37]. Their structure consists of two C6–C3 units linked by β,β’ bonds.

Lignans are one of the two main groups of phytoestrogens, which are well known for their good antioxidant properties. In fact, some antioxidant phytochemical compounds could be used as anticancer drugs as they are mimicking the functions of human hormones. Some studies on rats showed that lignans prevent the growth of breast and prostate tumors [38,39].

Numerous lignans could be considered as possible anticancer medicines due to their large pharmacologically valuable properties. Among all of them, arctigenin, magnolol, and honokiol are the main lignans investigated in medicine. Nonetheless, etoposide is a commercial lignin belonging to the podophilotoxin subfamily that is used in the treatment of different types of cancer such as lung cancer and breast cancer [40,41]. However, etoposide chemotherapy presents several side effects: low blood cell counts, vomiting, diarrhea, fever, loss of appetite, and alopecia.

#### 3.3.1. Arctigenin

Certain plants belonging to the family known as Compositae produce arctigenin, especially the seeds of greater burdock (*Arctium lappa*).

Some studies revealed that arctigenin inhibits the growth of various cancer cells: stomach, lung, liver, and colon, as well as leukocytes [42]. At the same time, the addition of arctigenin intensifies the activity of caspase-3, which is a protein that plays a crucial role in the death of carcinogenic cells. As a matter of fact, *Huang* et al. demonstrated that the treatment of OVCAR3 and SKOV3 ovarian cancers with arctigenin causes the apoptosis of cancer cells in vitro [43].

One of the most used conventional anticancer drugs is doxorubicin, which is a medicine that belongs to the anthracycline family applied in the treatment of, among other cancers, bladder, stomach, ovaries, lung and thyroid cancers. However, doxorubicin exhibits side effects among which the most frequent are severe nauseas, vomiting, and alopecia [44].

Studies were conducted by Lee et al. on adding natural products such as arctigenin to doxorubicin and determining the efficiency of both drugs in improving breast cancer treatment and reducing the side effects provoked by doxorubicin [45]. The work concludes that the combination of arctigenin and doxorubicin induced the apoptosis of MDA-MB-231 human breast cancer cells in vitro. The addition of arctigenin ameliorates the cellular uptake of doxorubicin, which causes the death of carcinogenic cells.

#### 3.3.2. Magnolol

Another lignan that was tested in some studies on cancer therapy is magnolol. As its name indicates, magnolol is an isomer of honokiol found in magnolia bark [46]. Since ancient times, extracts from the bark of magnolia have been used in traditional Chinese, Korean, and Japanese medicine.

In the last decades, the research on the use of natural products in various cancer treatments has been focused on attempts of understanding mechanisms that induce the antitumor agents’ response in the tumor cells [47]. This year, Su and co-workers elucidated the mechanism that reduces the endogen activity of nuclear factor kappa-light-chain-enhancer of activated B cells (NF-κB), which is a protein complex that controls DNA transcription and cell survival. Therefore, the cells that do not have regulated NF-κB can contribute to the onset and growth of various types of cancers.

Moreover, magnolol used in the treatment of colorectal cancer reduces the phosphorylation of protein kinase C delta type (PKCδ) and NF-κB, which are two proteins that are involved in tumour progression in vitro and in vivo [48].

Following the methodology used with other drugs, magnolol was co-encapsulated with trastuzumab, an anticancer drug commonly used in stomach or throat cancer therapies, and gold nanoparticles, building a nanocarrier cluster. The synthesized nanocarriers induced a specific photothermal near-IR response combined with targeted anticancer activity resulting in an improvement of magnolol cytotoxicity to breast cancer cells [49].

#### 3.3.3. Honokiol

As mentioned before, honokiol (also known as houpa or hnk) is a lignan isolated from the bark, seed cones, and leaves of trees belonging to the genus of magnolias, which includes around 210 species. Honokiol, which has been used in traditional eastern herbal medicines as an analgesic and together with magnolol, obovatol, and 4-*O*-methylhonokiol in the treatment of anxiety and mood disorders, has a spicy odor [46]. Honokiol is most frequently taken orally.

In nature, honokiol and magnolol isomers are found together. Usually, the separation and purification of both compounds had always been complexed, and it is commonly limited to HPLC. In 2006, Amblard and co-workers developed a method in which the authors protect the near hydroxyl groups in magnolol to produce a magnolol acetonide that can be simply separated from honokiol via flash chromatography over silica [50].

Recent studies suggest that honokiol could be an effective agent in cancer treatment due to its physical properties—honokiol’s ability to easily cross the blood–brain barrier and the blood–cerebrospinal fluid barrier—and its high bioavailability. Many research studies have shown that honokiol can kill carcinogenic cells in melanoma, sarcoma, myeloma, and leukemia, as well as in bladder, lung, prostate, and colon cancers [51,52].

Besides, honokiol enhances the apoptotic effects of some etoposides, such as doxorubicin. For instance, micelles with encapsulated doxorubicin and honokiol allow a controlled drug co-delivery that inhibits the progression of breast cancer tumors and reduces the doxorubicin side effects when compared with the micelles without honokiol [53].

On the other hand, the effectiveness of honokiol in the fight with typically drug-resistant multiple myelomas and chronic B-cell leukemia has been proved by various authors [54,55]. Ishitsuka and co-workers certified that honokiol presents the ability to kill drug-resistant multiple myeloma carcinogenic cells by varied mechanisms [56].

### 3.4. Phenolic Acids

Another subgroup of polyphenols that can be found in several plants, especially in dried fruit, are phenolic acids. These compounds are characterized by containing a phenolic ring and an organic carboxylic acid function (C6–C1 skeleton) [57]. Phenolic acids are divided in two classes:

- Derivatives of benzoic acid, and
- Derivatives of cinnamic acid.

In general, derivatives of cinnamic acid are more common in plants than the derivatives of benzoic acid. Despite that, some red fruit, onions, and black radish contain significant amounts of benzoic acid derivatives [58].

To date, the phenolic acid that exhibited medicinal properties that turn it into a plausible candidate for cancer treatment is *p*-coumaric acid.

#### *p*-Coumaric Acid

*p*-Coumaric acid (or 4-hydroxycinnamic acid) is an organic compound derived from cinnamic acid that can be found in a wide variety of edible plants (tomatoes, carrots, garlic, mushrooms, white beans, and others). Moreover, *p*-coumaric acid found in pollen is a constituent of honey [59].

Additionally, *p*-coumaric can be synthesized from cinnamic acid or L-tyrosine by the action of 4-cinnamic acid hydroxylase (C4H) or tyrosine ammonia lyase (TAL) enzymes, respectively.

During the last decade, few studies that evidenced the anticancer activity of *p*-coumaric acid in colon and gastric cancer cells have been published [60,61]. Lately, Jang et al. have shown that *p*-coumaric acid suppresses the growth of SNU-16 gastric cancer cells [62].

### 3.5. Flavonoids

**Figure 3:**
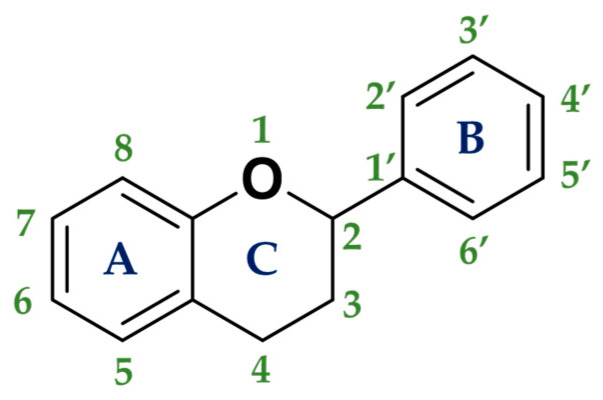
General structure of flavonoids.

The most important group of polyphenols are flavonoids. The chemical structure of flavonoids is composed of 15 carbon atoms comprising 2 cycles of six carbon atoms linked by a 3-carbon chain (rings A and B, in Figure 3). The flavonoids family consists of over 6000 molecules that have been identified and isolated, but there are undoubtedly many more flavonoid structures to discover [63].

Flavonoids are found in abundance in colored vegetables (spinach) and fruit such as berries, blueberries, apples, grapes, oranges, strawberries, plums, and in some foods and beverages widely used in the human diet, including dark chocolate, nuts, red wine, tea, soy, and soy derivatives.

Flavonoids have a wide spectrum of functions in plants:

- Flavonoids attract pollinating insects through the color or smell that they give to the plant or its flowers,
- Filtration of UV light,
- Protection against herbivorous predators,
- Protection against fungi,
- They are involved in the hormone auxin transport,
- Regulation of the cell cycle,
- Pigmented blue colors given by anthocyanins are responsible for the resistance of plants to the photooxidation of UV light from the sun, and
- In carnivorous plants, they attract prey.

Usually, two criteria are used to classify flavonoids:

- The chemical structure of the C heterocycle (if it is present), and
- To which carbon of the C ring the B ring is attached (C2 and C1′ in Figure 3).

**Figure 4:**
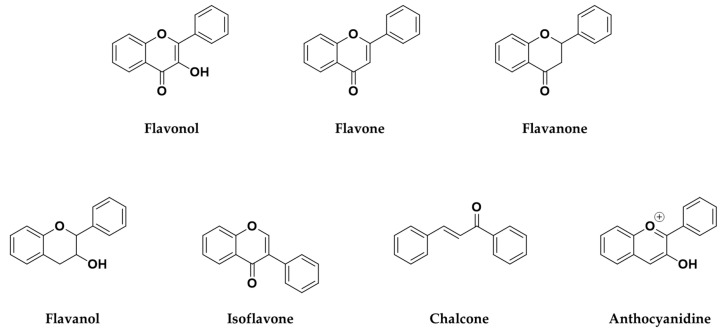
Chemical structure of the major subclasses of flavonoids.

According to these two factors, seven groups of flavonoids can be distinguished: flavonols, flavones, flavanones, flavan-3-ols, isoflavones, chalcones, and anthocyanidins (Figure 1). The chemical structures of these groups are shown in Figure 4.

**Figure 5:**
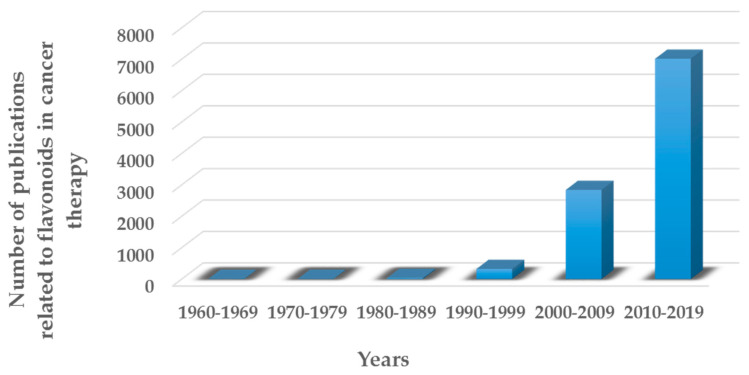
Number of peer-reviewed articles published in the last decades in the field of flavonoids in cancer therapy.

Figure 5 clearly demonstrates that the interest that flavonoids have attracted in recent years in terms of cancer treatment has grown rapidly [64].

#### 3.5.1. Flavonols

Flavonols are a class of flavonoids based on the backbone 3-hydroxyflavone. There is a wide variety of flavonols, which depend on positions that can be hydroxylated (Figure 4).

Many fruits (apples, peaches, oranges, blackberries, raspberries), vegetables (onions, broccoli, kale, Brussels sprouts, cucumbers, lettuce, tomatoes, potatoes, spinach), leaves (*Aloe Vera*, rosemary, soybean, *Pinus sylvestris*, holly, endive), seeds (grapes), and grains (several cereals including quinoa, buckwheat, barley, and oat) are rich sources of flavonols [65].

Flavonols are responsible for the color of flowers in some plants as well as protecting them from UV light and ROS [66].

Furthermore, flavonols are bioactive polyphenols that are widely used due to their excellent antioxidant properties [67]:

- In medicine: antimicrobial, anti-inflammatory, antiaging, anticancer, or insecticidal agents.
- In agriculture: as pesticides.

Kaempferol and quercetin are the main flavonols studied in medicine. Nevertheless, other flavonols such as herbacetin, myricetin, and fisetin have also been investigated as anticancer drugs [68,69].

##### Kaempferol

Kaempferol is a flavonol that is found in plants, plant-derived foods, and traditional medicines, including in tea, kale, beans, spinach, and broccoli [70]. Once isolated, kaempferol is a yellow crystalline solid of poor solubility. One study reported by Liu suggested that kaempferol intake contributes to approximately 17% of the total average intake of flavonols and flavones in a normal diet [71].

During the last few years, numerous investigations provided new evidence of the anticancer mechanisms of kaempferol both in vitro and in vivo. Discovering such mechanisms has enabled the analysis and understanding of kaempferol’s role as an anticancer drug and afterwards may lead to an improvement of applied techniques and methods, such as the development of kaempferol-loaded targeted drug delivery systems [72].

One of the cancers in which the effect of kaempferol has been studied the most is breast cancer [71]. Several research groups have proved the cytotoxicity of kaempferol against breast cancer cells both in vitro and in vivo:

- By inhibiting the growth of cancer cells,
- By stopping the progression and proliferation of cancer cells, and
- By inducing cancer cells apoptosis.

One of the latest investigations to clarify the mechanism of kaempferol as an anticancer drug against breast tumors was carried out by Zhu et al. The authors mentioned that kaempferol induced apoptosis and DNA damage in MDA-MB-231 cancer cells by the upregulation of the phosphorylated form of the H2A histone family member X (γH2AX), caspase 3, caspase 9, and the protein serine/threonine kinase (p-ATM) [73].

Da and co-workers tested kaempferol in prostate cancer cells [74]. The authors concluded that the use of kaempferol against LNCaP prostate cancer cell lines led to cancer cells death and impeded cancer cell proliferation and invasion in a dose-dependent manner.

##### Quercetin

Quercetin is the most common flavonoid in human diet with an average daily consumption of 25–50 milligrams [75]. Quercetin is mainly found in red onions, kale, apples, grapes, broccoli, and tea. In red onions, quercetin represents around 10% of its dry weight.

Various in vitro and in vivo studies showed that quercetin is one of the most potent antioxidants of the flavonoid family [76], which makes it an ideal candidate for an anticancer drug. Indeed, quercetin is the active ingredient of Yang-Yin-Qing-Fei-Tang, which is a traditional Chinese medicine. Furthermore, quercetin exhibited cytotoxicity in various tumor cells, in breast, cervical, colon, liver, lung, gastric, prostate cancers, and in leukemia [77,78].

Making use of the anticancer effects of quercetin, the most recent studies combined quercetin with other anticancer drugs with the aim of increasing the efficiency of cancer therapies. Some examples are summarized below.

One of the natural compounds that lately has been combined with quercetin in cancer therapy studies is curcumin. Srivastavaa et al. showed that the mixture of quercetin and curcumin improved the inhibition of cancer cell proliferation by regulating the Wnt/β-catenin signaling and promoting the carcinogenic cells death by distinct pathways [79].

Furthermore, Sunoqrot and co-workers combined both curcumin and quercetin by preparing nanoparticles with encapsulated curcumin and a shell of quercetin covalently bonded with polyethylene glycol (PEG) prepared in a one-pot procedure [80]. Once tested in vivo, these nanocarriers exhibited a controlled drug delivery of curcumin in physiological conditions, which makes it a potentially powerful tool in cancer therapy.

It has also been observed that the addition of quercetin to docetaxel therapy in prostate cancer reduces the docetaxel resistance of carcinogenic cells. That increases the efficacy of cancer therapy resulting from an intensification of the apoptosis of cancer cells and the reduction of tumor proliferation and migration [81].

#### 3.5.2. Flavones

**Figure 6:**
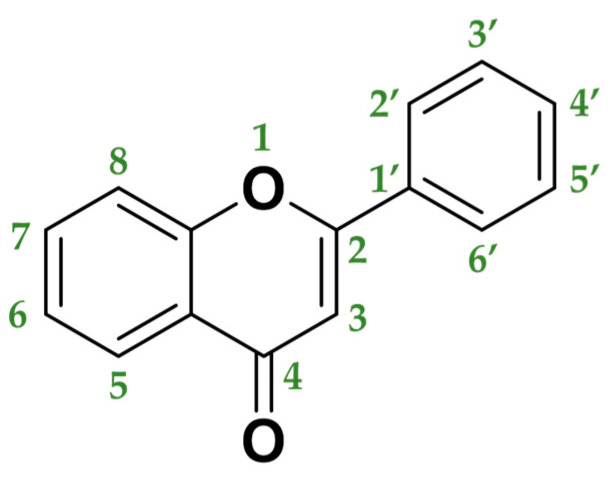
Molecular structure of flavone backbone.

Flavones are a class of flavonoids with a chemical structure very similar to flavonols, from which they only differ in the non-hydroxyl substitution at the carbon 3-position of flavones (Figure 6). Flavones are basically found in herbs (parsley, thyme, chamomile, mint, chrysanthemum flowers) and red or purple plants and vegetables (apple skins, broccoli, cabbages, celery, onion leaves, carrots, and red peppers) [82]. In plants, flavones usually act as defense mechanisms against diseases originated by pathogens.

Some of the flavones have been in use for many years. The most representative example is luteolin, which since ancient times has been used as yellow dye. Apigenin has also been used to dye wool. Moreover, wogonin is well known because it is one of the active ingredients of Sho-Saiko-To, which is a Japanese herbal supplement [83].

However, the interest in using this family of flavonoids in medicine has been growing because they demonstrate efficient antimicrobial, antioxidant, antifungal, anti-inflammatory, antimutagenic, and anticancer activity [84]. Inside the flavones family, the anticancer properties of apigenin and luteolin are widely investigated.

##### Apigenin

Apigenin, which is a yellow crystalline solid, is one of the flavones most commonly found in nature. Many fruits and vegetables, such as parsley, celery, celeriac, carrot, oregano, and chamomile tea contain apigenin. In the particular case of chamomile tea, apigenin constitutes 68% of the total flavonoids content [85].

For many centuries, apigenin has been widely used as a traditional medicine [86,87]. The excellent properties of this natural compound have prompted the study of its application as an anticancer drug [88,89]. In fact, various positive effects of apigenin administration, alone or in combination with other chemotherapeutic agents, in different types of cancer treatments were reported in the literature [90]. The following aspects were mentioned:

- Inducing the death of cancer cell lines,
- Triggering both autophagy and apoptosis,
- Suppressing cancer cell migration and invasion, and
- Inducing the cancer cells cycle arrest.

One of the recently carried out investigations mentions that apigenin promotes pancreatic cells death by increasing intracellular ROS [91]. In this work, Montani et al. tried to understand the mechanism happening in cancer cells in which apigenin was applied. In fact, they suggested a biological mechanism occurring between heat shock protein (HSP90), a protein that stabilizes proteins involved in the growth of cancer cells, and TP53 gene mutations that reduce the cytotoxic effect of the chemotherapy with apigenin. The targeting of these molecules is an important anticancer strategy that has been extensively explored.

On the other hand, Liu et al. evaluated the synergistic effect in cancer therapy involving apigenin combined with metal ions [92]. In this work, the authors examined the thermal stability of two flavones (apigenin and luteolin) when combined with ferrous or cupric ions, which negatively affects the anticancer activities of both flavones against human cervical cancer Hela cells.

##### Luteolin

Luteolin is usually found in the leaves and bark of some plants. The major natural sources of luteolin are celery, thyme, dandelion, clover flower, ragweed pollen, chamomile, and perilla [93].

Due to its beneficial effects on the human body (antioxidative and anti-inflammatory properties, being a free radicals scavenger, promoting carbohydrate metabolism, and modulating the immune system), it is assumed that luteolin could perform an important role in cancer therapy [94,95].

To enhance the anticancer effects of luteolin, the flavone is usually used together with other anticancer drugs. Ren and co-workers demonstrated that the application of luteolin in combination with oxalipatlin, a conventional anticancer drug used to inhibit the development of cancer cells, stopped the proliferation of gastric cancer cells in vitro by the upregulation of the activity of caspase-3 and Bax proteins [96].

The construction of nanocarriers containing anticancer drugs allows obtaining controlled drug delivery systems. By the encapsulation of luteolin in polymeric micelles, Hu et al. developed a thermosensitive nanocarrier that demonstrated an improved apoptosis of colorectal cancer cells compared to the administration of free luteolin [97].

#### 3.5.3. Flavanones

Flavanones are colorless ketones derived from flavone. Flavanones are found in a wide variety of foods included in our daily diet and in herbs [82,98]:

- Fruits: orange, lemon, lime, tangelo, grapefruit (especially in citrus fruits), strawberry, raspberry, plum.
- Vegetables: tomato, potato.
- Herbs: rosemary, peppermint.

In citrus fruits, flavanones are usually glycosylated by a disaccharide in position 7 (Figure 3).

They present different functions in plants:

- Antioxidative (Pinocembrin),
- Antimicrobial (Sakuratenin),
- Taste-modifying properties (Eriodictyol, homoeriodictyol and sterubin), and
- They are responsible for the bitter taste in citrus fruits (Naringin).

In the last decades, flavanones have gained a lot of importance in medicine for their antioxidant activity, radical scavenging, cardiovascular, anti-inflammatory, antiviral, and anticancer effects [99]. Naringenin and hesperetin are the most often investigated for being anticancer drugs. Nevertheless, some tests were carried out using other flavanones such as didymin and alpinetin [100,101].

##### Naringenin

Naringenin is a flavanone predominating in oranges and grapefruits. It is also found in bergamot, sour orange, tomatoes, cocoa, water mint, beans, etc. [102,103]. In some of these fruits, narigenin is present in its glycosidic form: naringin (which has attached a disaccharide neohesperidose via a glycosidic linkage at carbon 7).

As it has been proven in several studies, naringenin induces cytotoxicity in various carcinogenic cells of breast, stomach, liver, cervix, pancreas, colon cancers, and in leukemia [104]. Nevertheless, its poor solubility and instability in physiological medium limits the medical applications of naringenin. To solve these drawbacks, Akhter et al. reported the encapsulation of naringenin in PLGA (poly(lactide-co-glycolid acid)) nanoparticles. Moreover, they suggested that the encapsulated naringenin showed higher cytotoxicity when compared with free naringenin due to a more controlled drug release [105]. Another option that could enhance the anticancer properties of naringenin involves the synthesis of naringenin derivatives [106].

An alternative recent study demonstrated naringenin’s effectivity as an anticancer drug in breast cancer treatment is due to the activation of the caspase-3 protein and caspase-9 enzymes [107], while Kumar and co-workers showed in vivo that naringenin showed antitumor effects on skin cancer [108].

##### Hesperetin

Hesperetin and hesperetin’s 7-*O*-glycoside (also known as hesperidin) are the main flavonoids found in lemons and sweet oranges [109].

Hesperetin’s anticancer properties against specific tumors are well documented in numerous research publications:

- It inhibits glucose uptake in various cancer cell lines [110,111],
- Reduces the NF-κB activity, which leads to a decrease in tumor progression [112], and
- Upgrades the apoptosis via the induction of intracellular ROS formation [113].

In a more recent study, the addition of hesperetin improves the activity of cisplatin, which is an anticancer drug that is commonly used to treat lung cancer [114]. It was observed that hesperetin inhibits MDR protein (multidrug resistance protein 1), which is associated with the resistance to cisplatin developed in a great number of patients subjected to cancer therapy.

Curiously, the administration of both naringenin and hesperetin were tested in vitro and in vivo trials to analyze the anticancer effects in human pancreatic cancer [115]. For the first time, the authors reported that the combination of both naringenin and hesperetin could be used as a potential non-toxic cancer therapy system that stops pancreatic cancer development.

#### 3.5.4. Flavanols

Flavanols or flavan-3-ols are another group of monomeric flavonoids. Catechin and its derivatives are included in this group. Natural sources of flavan-3-ols are mainly the “tea plant” (*Camellia sinensis*), and some cocoas. Therefore, they are highly present in the human diet in both beverages (tea) and solid foods (chocolates) [82,98].

Since studies of flavanols have started in the course of the last century, it has been found that these compounds provide resistance against dangerous trespassers, including microbes, fungi, insects, and herbivorous animals [116].

Thereby, the flavanols’ health benefits have been broadly studied in humans. Some investigations suggest that the intake of cocoa flavanols could help in the prevention of cardiovascular and metabolic diseases. Indeed, the European Food Safety Authority approved cocoa products containing 200 mg of flavanols because they “help to maintain the elasticity of blood vessels, which contributes to normal blood flow” [117].

##### Epigallocatechin Gallate

Epigallocatechin gallate (epigallocatechin-3-gallate or EGCG) is a catechin that is mostly found in tea and one of the polyphenolic compounds most commonly found in nature; it is also the ester of epigallocatechin and gallic acid [118].

The objective of finding a correlation between green tea intake and the risk of cancer onset has been a well-studied topic [119]. As an obvious example, the study presented by Guo et al. [120] validated that the consumption of green tea—and therefore catechins—up to seven cups a day provided a small reduction in the prostate cancer risk.

Moreover, EGCG has been tested against certain cancer cell lines. In HT-29 colorectal cell lines, EGCG upregulated the activity of TfR (Transferrin receptor), which is a carrier protein for transferrin, and inhibited the activity of the Ferritin-H protein via the iron chelation activity in HT-29 colorectal cancer cells [121]. In another example, the synergistic effect of EGCG and TRAIL (Tumor necrosis factor (TNF)-related apoptosis-inducing ligand), a protein that causes cell death, intensifies the activity of both caspase 8 and the death receptor 5, causing the death of SW480 and HCT116 colon cancer cells [122].

Despite the fact that EGCG is commonly found in nature, this flavanol shows some drawbacks that limit its applications in cancer therapy (poor stability, low absorption, and hepatotoxicity) [123]. So, the encapsulation of EGCG can be a promising solution to minimize the limitations of the EGCG use [124].

##### (−)-Epicatechin

The (−)-epicatechin molecule is a flavonoid of which large quantities are found in cocoa [125]. The use of epicatechin in cancer therapy has been emerging over the last decade in the attempt to overcome some of the drawbacks of EGCG [126,127].

Pereyra-Vergara and co-workers studied the effects and mechanism of (−)-epicatechin in breast cancer cells [128]. It was shown that the addition of (−)-epicatechin to carcinogenic cells results in the apoptosis of the two tested breast cancer cell lines (MDA-MB-231 and MCF-7). Moreover, the authors proved that (−)-epicatechin increased the intracellular ROS production and intensified the activity of BCL2 associated agonist of cell death (Bad) and bcl-2-like protein 4 (Bax), proteins that are associated with cell apoptosis.

#### 3.5.5. Isoflavones

Isoflavones are another type of biological active flavonoids. Isoflavones are mostly found in plants of the leguminosea family. This family includes many species that are of great importance in the human diet (peas, lentils, licorice, beans, chickpeas, and carob), in animal fodder (alfalfa, clover, and carob) and as ornamental plants (mimosa and false acacia) [82,129].

Since isoflavones present estrogenic properties, plants use these kinds of compounds as part of their natural defense system against the overpopulation of herbivores by controlling their male fertility [130]. Moreover, these properties make isoflavones good complementary therapeutic options in treating menopause and its symptoms such as osteoporosis, anxiety, emotional instability, and headaches. Genistein and daidzein are the most studied compounds of this subgroup in terms of medical applications. Nevertheless, other isoflavones such as glabridin and alpinumisoflavone have raised interest as potential cancer medicines in various types of cancer such as breast, liver, or thyroid cancers [131,132].

##### Genistein

The isoflavone most reported in medicine is genistein, which is a phytoestrogen compound produced in soybeans. Genistein was for the first time isolated in 1899. However, it was not until the end of the last century that researchers started to explore its potential beneficial effects on human health and its possible applications as a medical compound in a wide range of diseases, including cardiovascular diseases, osteoporosis prevention, diabetes, and some types of cancers [133]. It has been proven that genistein is involved in the regulation of different genes that are associated with the onset of cancers by various mechanisms [134].

In a recent research, Hsiao et al. studied the effects and mechanisms of genistein against leukemia cell lines. In fact, the application of genistein to HL-60 leukemia cells revealed that this natural medicine kills the carcinogenic cells via two different pathways (endoplasmatic reticulum stress and mitochondria-dependent pathway) in vitro and in *mouse xenograft* models in vivo [135].

Furthermore, different authors studied the effects of genistein when it is combined with other anticancer drugs [136]. In a recent investigation, Liu et al. tested mixtures of genistein and cisplatin in varied concentrations as a plausible anticancer agent in the treatment of cervical cancer cells [137]. The authors proved that the addition of genistein improved the chemotherapeutic activity of cisplatin, requiring a lower dose of the drug in cancer treatment, which led to a reduction in the therapy side effects.

##### Daidzein

The second isoflavone most commonly found in nature, which similar to genistein is also isolated from soybeans, is daidzein [138]. The chemical structure of daidzein is very similar to genistein, without the hydroxyl group at position 5 (Table 1).

Rigalli et al. studied in vitro the effects of daidzein use in breast cancer therapy [139]. In one of those studies, they proved that daidzein downregulated the expression of multidrug resistance-associated protein 1 (MRP1) in both Michigan Cancer Foundation-7 (MCF-7) and MDA-MB-231 breast cancer cell lines. The reduction of this protein’s activity is very important because MRP1 is involved in transporting many of the chemotherapeutic drugs out of the cells (for example, doxorubicin or mitoxantrone).

In another study in vivo, mice were inoculated with 4T1 breast cancer cells and then treated with daidzein administered orally for 22 days. In this case, the highest dose of daidzein (145 mg/kg) was required to observe a considerable decrease in tumor size. At the same time, the authors reported that the combination of daidzein with regular exercise promotes the breast cancer cells apoptosis via the Fas/FasL-mediated mechanism [140].

#### 3.5.6. Chalcones

Chalcones are a class of polyphenolic compounds that are characterized by the presence of an aromatic ketone and an enone in their central core. Many fruits such as citrus and apples, vegetables such as tomatoes, potatoes, shallots, and bean sprouts, and some edible plants such as licorice contain chalcones [141].

Besides, chalcones can be synthesized in the form of base-catalyzed aldol condensation of benzaldehydes with acetophenones (for example, sodium hydroxide) [142].

The most studied chalcone in the field of medicine is ellagic acid, which has been investigated as a potential antitumor agent [143,144].

##### Ellagic Acid

Ellagic acid is an antioxidant that is found in various natural resources: in oak species such as white oak (Quercus alba) and European red oak (*Quercus robur*) or in medicinal fungi (*Phellinus linteus*). Peaches, pomegranates, grapes, strawberries, raspberries, pecans, walnuts, and raw chestnuts also contain a considerable amount of ellagic acid [145].

The anti-proliferative and antioxidative properties of ellagic acid have encouraged researchers to study the health benefits of this natural compound. For years, the effects of treating tumors with ellagic acid have been studied by the evaluation of various alternatives (chemical modifications of ellagic acid or its encapsulation among other options) [146].

One of the last studies that examined the breast cancer treatment with ellagic acid was published by Yousuf et al. [147]. This work evaluated the capacity of numerous phytochemicals in addition to ellagic acid (capsaicin, tocopherol, rosmarinic acid, ursolic acid, limonene, caffeic acid, and ferulic acid) to inhibit the activity of cyclin-dependent kinase 6 (CDK6), which is an important gene associated with cancer progression. Among all the tested natural compounds, ellagic acid showed the highest binding affinity for CDK6, decreasing the tumor proliferation.

However, the encapsulation of ellagic acid to enhance its poor solubility combined with an improvement of its controlled delivery was attempted by some research groups [148,149]. In a recent work, *Pirzadeh-Naeeni* et al. reported the nanoencapsulation of ellagic acid in two different biopolymers (schizophyllan and chitin), which were then tested against MCF-7 breast cancer cells [150]. In this case, the controlled release of ellagic acid improved the cytotoxicity when compared with non-encapsulated ellagic acid. It also reduced the progression of tumor cells.

#### 3.5.7. Anthocyanidins

Anthocyanidins are water-soluble pigments found in plants. They are responsible for leaves, flowers, and fruit colors. Some fruits included in the human diet are rich in anthocyanins: blueberries, raspberries, black rice, and black soybeans (normally known as dark fruit). The term anthocyanin was coined in 1835 by Ludwig Clamor Marquart, a German pharmacist, to denote the blue pigment of red cabbage (*Brassica oleracea*) [151].

Anthocyanidins have varied functions in plants: attracting pollinating insects, preventing the freezing of fruits such as grapes, and protecting plants against harmful UV radiation [152].

Moreover, these kinds of compounds are widely used in the food industry (preparation of food coloring, a parameter for determining wine quality) and in medical industry (decreased risk of contracting various diseases such as obesity, improved memory and age-related deficiencies, or improvement of the immunological system) due to their chemical and physical properties [153].

Some anticancer properties of anthocyanidins extracted from the plant *Cyanomorium coccineum* have been recently described by *Rescigno et al.*, which demonstrated the antiproliferative effect of anthocyanidins against different leukemia cell lines [154].

##### Delphinidin

Delphinidin, which can be found in red cabbage, grapes, berries, and sweet potatoes among other colored fruits and vegetables, is one of the most common anthocyanidins [155,156].

The antitumour activity of delphinidin has been demonstrated by numerous researchers. In 2016, Jeong et al. studied the effect of delphinidin in prostate cancer treatment. They found that delphinidin increased the activity of caspase-3, -7, and -8, in effect causing the death of cancer cells. Moreover, they demonstrated that delphinidin intensified the roles of genes that induce the apoptosis of cancer cells and decreased the activity of some genes that dissuade killing the cancer cells [157].

Alternatively, delphinidin obstructs the progression of SKOV3 ovarian cancer cells in vitro by decreasing the Akt Pathway (a signal transduction pathway) activation, which can in result activate numerous factors that play a critical role in cancer migration [158].

## 4. Overview

The chemical structure of the polyphenolic compounds mentioned in this review, their anticancer effects, and the corresponding references are summarized in Table 1. As indicated in the table, the administration of resveratrol and quercetin has been approved by the Food and Drug Administration (FDA).

## 5. Conclusions

In conclusion, the exceptional antioxidative properties make polyphenols strong candidates for agents used in various types of cancer treatments. Actually, the anticancer effects of several polyphenolic compounds have been mainly studied in in vitro cancer cells and in preclinical animal models.

Nevertheless, there are very few clinical data on many of the polyphenols application as anticancer medicines (clinical studies on cancer therapy involve only the most common polyphenols such as resveratrol, curcumin, and quercetin). Nowadays, the vast majority of these clinical studies are still in progress.

The research on cancer therapies involving varied polyphenol families, and particularly flavonoids, has contributed to the development of natural medicines that are less aggressive than conventional anticancer drugs. In fact, various research works proved that polyphenols could be used as chemotherapy adjuvant agents in cancer therapies.

However, the process of discovering the polyphenols’ mechanisms of action as anticancer drugs and their interactions with tumors requires further studies in order for those natural compounds to improve the actual therapeutic strategies.

## Figures and Tables

**Table 1:** Summary of various polyphenols, their chemical structures, and their anticancer effects.

Polyphenol	Applications in Cancer Therapy	References
Resveratrol	DNA protection against reactive oxygen species (ROS), trap the hydroxyl and superoxide groups and the free radicals produced into the cells. Inhibition of A549 lung cancer cells with the activation of Caspase-3. U. S. Department of Health and Human Services Public Health Service Food and Drug Administration Status: Bulk ingredient for human prescription compounding.	[23,24]
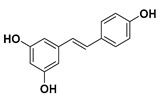		
Curcumin	Reduce the expression of survivin and promotes E-chaderin. Apoptosis of colon cancer cells. Synergistic effects of curcumin and resveratrol in lung and prostate cancer.	[34,35,36]
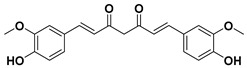		
Arctigenin	Inhibition of surviving and inducible Nitric oxide synthases (iNOS) expression and activation of caspase-3 protein. Apoptosis of OVCAR3 and SKOV3 ovarian cancer cells. Improves the cellular uptake of doxorubicin and reduces its side effects. Apoptosis of MDA-MB-231 breast cancer cells.	[41,43]
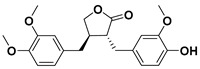		
Magnolol	Induces apoptosis of colorectal cancer cells through extrinsic/intrinsic pathways and inhibits nuclear factor kappa-light-chain-enhancer of activated B cells (NF-κB) signaling through protein kinase C delta type (PKCδ) inactivation. Upgrades the cytotoxicity of trastuzumab and increases the specificity to breast cancer cells.	[46,47]
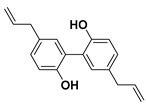		
Honokiol	Synergistic effects of honokiol and doxorubicin in breast cancer by suppressing the metastasis of carcinogenic cells and apoptosis induction. Apoptosis of multiple myeloma cancer cells.	[49,52]
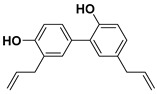		
*p*-Coumaric acid	Apoptosis of HCT-15 colon cancer cells through ROS mitochondrial pathway. Inhibits the growth of SNU-16 gastric cancer cells.	[56,57,58]
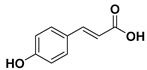		
Kaempferol	Induces the apoptosis and DNA damage in MDA-MB-231 breast cancer cells by the upregulation of H2A histone family member X (γH2AX), caspase 3, caspase 9, and the protein serine/threonine kinase (p-ATM). Induces the apoptosis of LNCaP prostate cancer cells. Impedes the proliferation of cancer cells.	[69,70]
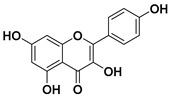		
Quercetin	Synergistic effects of quercetin and curcumin: Inhibition of cancer cell proliferation by regulation of the Wnt/β-catenin signaling and apoptotic pathways. Reduces docetaxel resistance effect in prostate cancer cells. U. S. Department of Health and Human Services Public Health Service Food and Drug Administration Status: Drug for further processing.	[75,77]
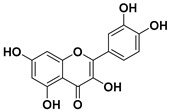		
Apigenin	Promotes apoptosis of pancreatic cancer cells by increasing intracellular ROS. Damages DNA of Hela cervical cancer cells. Inhibits the growth of cancer cells and induces its apoptosis.	[87,88]
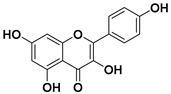		
Luteolin	Synergistic effects of luteolin and oxaliplatin: stops the proliferation of gastric cancer cell. Promotes apoptosis and stops the proliferation of colorectal cancer cells.	[92,93]
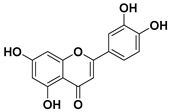		
Naringenin	Apoptosis of breast cancer cells by the increase of the activity of caspase-3 and caspase-9. Suppression of skin cancer cells.	[103,104]
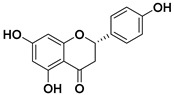		
Hesperetin	Synergistic effects of hesperetin and cisplatin: Modulation of the nuclear factor kappa-light-chain-enhancer of activated B cells (NF-κB) signaling pathway in A549 lung cancer cells. Inhibition of the multidrug resistance protein 1 (MDR 1) protein. Increases cisplatin efficiency. Synergistic effects of hesperetin and naringenin: inhibition of the growth of pancreatic cancer cells.	[110,111]
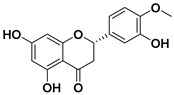		
EGCG	Upregulates the activity of transferrin receptor (TfR) and inhibits the activity of Ferritin-H protein via iron chelation activity in HT-29 colorectal cancer cells. Synergistic effects of epigallocatechin-3-gallate (EGCG) and tumor necrosis factor (TNF)-related apoptosis-inducing ligand (TRAIL): Increases caspase 8 activity and suppresses receptor 5. Apoptosis of SW480 and HCT116 colon cancer cells.	[117,118]
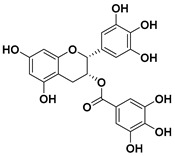		
(−)-Epicatechin	Increasing intracellular ROS and the activity of BCL2 associated agonist of cell death (Bad) and bcl-2-like protein 4 (Bax) proteins, which results in the apoptosis of MDA-MB-231 and Michigan Cancer Foundation-7 (MCF-7) breast cancer cells.	[124]
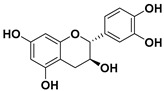		
Genistein	Apoptosis of HL-60 leukemia cancer cells via endoplasmatic reticulum stress and mitochondria-dependent pathways. Synergistic effects of genistein and cisplatin: Improves the chemotherapeutic activity of cisplatin.	[131,133]
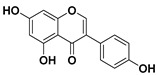		
Daidzein	Decreases the expression of the multidrug resistance-associated protein 1 (MRP1) protein in both MCF-7 and MDA-MB-231 breast cancer cells. Synergistic effects of daidzein and doing regular exercise: promotes the apoptosis of 4T1 breast cancer cells via the Fas/FasL-mediated mechanism.	[135,136]
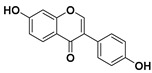		
Ellagic acid	Inhibits cyclin-dependent kinase 6 (CDK6) gene activity. Decreases tumor proliferation in breast cancer cells. Cytotoxicity against MCF-7 breast cancer cells. Decreases tumor progression.	[143,146]
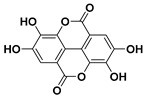		
Delphinidin	Increases the activity of caspase-3, -7, and -8, causing the death of LNCaP prostate cancer cells. Intensifies the roles of genes involved in cancer cell apoptosis. Reduces the activity of genes that dissuade killing cancer cells. Obstructs the progression of SKOV3 ovarian cancer cells by decreasing the Akt activation.	[152,153]